# Lead Exposure Inhibits Fracture Healing and Is Associated with Increased Chondrogenesis, Delay in Cartilage Mineralization, and a Decrease in Osteoprogenitor Frequency

**DOI:** 10.1289/ehp.7596

**Published:** 2005-03-14

**Authors:** Jonathan J. Carmouche, J. Edward Puzas, Xinping Zhang, Prarop Tiyapatanaputi, Deborah A. Cory-Slechta, Robert Gelein, Michael Zuscik, Randy N. Rosier, Brendan F. Boyce, Regis J. O’Keefe, Edward M. Schwarz

**Affiliations:** ^1^Center for Musculoskeletal Research, University of Rochester Medical Center and; ^2^Department of Environmental Medicine, University of Rochester, School of Medicine and Dentistry, Rochester, New York, USA

**Keywords:** endochondral ossification, fracture healing, lead, osteoblast, osteoclast, Pb, Pb toxicity

## Abstract

Lead exposure continues to be a significant public health problem. In addition to acute toxicity, Pb has an extremely long half-life in bone. Individuals with past exposure develop increased blood Pb levels during periods of high bone turnover or resorption. Pb is known to affect osteoblasts, osteoclasts, and chondrocytes and has been associated with osteoporosis. However, its effects on skeletal repair have not been studied. We exposed C57/B6 mice to various concentrations of Pb acetate in their drinking water to achieve environmentally relevant blood Pb levels, measured by atomic absorption. After exposure for 6 weeks, each mouse underwent closed tibia fracture. Radiographs were followed and histologic analysis was performed at 7, 14, and 21 days. In mice exposed to low Pb concentrations, fracture healing was characterized by a delay in bridging cartilage formation, decreased collagen type II and type X expression at 7 days, a 5-fold increase in cartilage formation at day 14 associated with delayed maturation and calcification, and a persistence of cartilage at day 21. Fibrous nonunions at 21 days were prevalent in mice receiving very high Pb exposures. Pb significantly inhibited *ex vivo* bone nodule formation but had no effect on osteoclasts isolated from Pb-exposed animals. No significant effects on osteoclast number or activity were observed. We conclude that Pb delays fracture healing at environmentally relevant doses and induces fibrous nonunions at higher doses by inhibiting the progression of endochondral ossification.

Despite stringent governmental regulations, lead exposure continues to be a major public health problem, with blood levels being elevated in a bimodal distribution predominantly affecting young children 1–5 years of age and individuals > 50 years of age (Pirkle et al. 1998). The Centers for Disease Control and Prevention’s (CDC’s) Childhood Lead Surveillance Program monitors blood Pb at the state and local levels. Mean national blood Pb levels have decreased dramatically over the past 30 years, as documented by the third National Health and Nutrition Examination Survey, Phase 2 (NHANES, 1991–1994) (CDC 2000; Pirkle et al. 1998). According to NHANES data from 1999 and CBLS data from 1996–1998, despite the decreases in mean national blood Pb among children, levels remain elevated in specific areas, affecting mostly low-income, urban children and those living in older housing (CDC 2000).

In addition to children, many adults maintain elevated blood levels due to past occupational or environmental exposure. With the reduction of Pb in fuel and soldered cans as well as increased awareness and vigilance, acute environmental Pb exposure has decreased dramatically (Pirkle et al. 1998). However, Pb becomes sequestered in the skeleton, incorporated into hydroxyapatite crystals during calcification, and remains there until the bone is resorbed or remodeled (Wittmers et al. 1988).

Elevated blood Pb levels, particularly perimenopausal, may have a causative role in the pathogenesis of the costly metabolic bone disease osteoporosis. Blood Pb levels increase during periods of high bone turnover such as menopause (Silbergeld et al. 1988, 1993). Additionally, the aging process itself has been shown to increase the release of Pb from the skeleton by cadaveric analysis as well as by experimental study (Barnes et al. 1999). *In vivo* models have demonstrated a decrease in bone density with Pb exposure (Escribano et al. 1997; Gruber et al. 1997). In addition, multiple reports in humans and animals support a role for Pb in osteopenia (Berlin et al. 1995; Silbergeld et al. 1988). The decrease in bone quality may not only cause individuals to cross a fracture threshold earlier, but, we hypothesize, it may also impede normal fracture healing.

It is widely appreciated that the role of the skeleton in Pb toxicokinetics is greater than that of a reservoir. Several authors have described the inverse relationship between elevated blood Pb levels and skeletal development, chest circumference, and stature (Pearl and Boxt 1980; Schwartz et al. 1986; Shukla et al. 1989). The effects on adults are more subtle. Previously, we identified chondrocytes as important targets of Pb toxicity (Puzas et al. 1992) and demonstrated that Pb suppresses the expression of phenotypic markers in growth plate chondrocytes (Hicks et al. 1996). More recently, we have shown that Pb alters the effects of parathyroid-hormone–related peptide, transforming growth factor-β (TGF-β), activator protein-1, and nuclear factor-κB signaling in chondrocytes (Zuscik et al. 2002). Histomorphometric studies have demonstrated a significant Pb-associated decrease in length of rat femoral growth plate cartilage (Gonzales-Riola et al. 1997). Together, these data suggest an inhibitory effect on endochondral ossification (Gonzales-Riola et al. 1997; Hicks et al. 1996).

Several authors have demonstrated adverse effects of Pb on both bone formation and resorption mediated by cellular pathways affecting osteoblasts and osteoclasts (Dowd et al. 1990; Long et al. 1990; Miyahara et al. 1994; Pounds et al. 1991; Schanne et al. 1989, 1990). Osteoblasts are known targets of Pb toxicity from *in vitro* studies with ROS 17/2.8 cells, which demonstrated suppression of alkaline phosphatase, type I collagen, and osteocalcin (Long et al. 1990). In addition, circulating levels of osteocalcin, which serve as markers of osteoblast activity and regulators of bone formation and remodeling (Ducy et al. 1996), are decreased in Pb-intoxicated children (Markowitz et al. 1988). The mechanism by which these effects occur remains unclear.

Fracture healing is a complex orchestration of several cell types. It is unique in that healing occurs by reformation of bone rather than scar tissue. Fracture healing involves primary recruitment, proliferation, and differentiation of both osteoprogenitors (osteoblast progenitors), for intramembranous ossification, and undifferentiated mesenchymal cells, for endochondral bone formation (Barnes et al. 1999; Einhorn 1998). Fracture healing is an established means of studying the formation and repair of skeletal elements *in vivo* as well as the crucial signaling pathways involved (Zhang et al. 2002).

Given the known effects on cells involved in bone formation and remodeling and the absence of literature on Pb in skeletal repair, the purpose of the present study was to evaluate the effects of elevated blood Pb levels on fracture healing. We employed an established murine tibia fracture model (Bonnarens and Einhorn 1984; Einhorn 1998) to characterize the effects of Pb on skeletal repair.

## Materials and Methods

### Pb exposure and whole blood Pb level determination.

All Pb solutions were made using Pb acetate (Gibco, Grand Island, NY) dissolved in distilled water. All mice had unrestricted access to the normal rodent diet supplied by the vivarium staff, and drinking water was replenished at least one time per week by the investigative staff to ensure continuous Pb exposure. All Pb waste solutions were disposed of appropriately. Mice were housed in groups of ≤5 animals per microisolator cage in the vivarium and maintained on a 12-hr light/dark cycle. All procedures were carried out in accordance with the regulations of and following the approval of the University of Rochester Animal Use and Care Committee.

Female C57/Bl6 mice (*n* = 32) at 6 weeks of age were divided into eight groups of four mice each. Each group was exposed to one of the following doses: 0, 55, 230, 580, 1,160, 1,750, 2,300, or 5,800 ppm Pb in the drinking water. This preliminary exposure was carried out to determine the whole-blood Pb (BPb) concentrations needed to approximate environmentally relevant levels of human exposure. This cohort is referred to as group A. Pb exposures of 0, 55, and 230 ppm Pb in the drinking water were selected as most environmentally relevant. A second cohort (group B) of female C57/Bl6 mice (*n* = 54) was divided into three groups of 18 animals and housed under conditions as outlined above. All experiments related to the analysis of the fracture healing process were performed with group B except one, which documented chronic nonunions in mice exposed to 2,300 ppm Pb.

Before Pb exposure, 100 μL whole blood samples were collected for baseline BPb level measurement. Samples were collected, using the saphenous vein technique (Hem et al. 1998), into 100 μL nitric acid–washed volumetric capillary tubes (VWR, Buffalo Grove, IL). Each whole-blood sample was then immediately diluted 1:10 with a matrix modifier solution containing 0.2% NH_4_H_2_PO_4_, 0.5% Triton X-100, and 0.2% HNO_3_ (Parsons 1993). Whole blood was collected for BPb analysis at 3-week intervals.

BPb levels were determined using a Perkin-Elmer A Analyst 600 atomic absorption spectrophotometer equipped with longitudinal Zeeman background correction and a transverse heated graphite furnace (PerkinElmer Life and Analytical Sciences, Boston, MA). This method was adapted by Parsons from the Department of Environmental Health and Toxicology, SUNY at Albany (Parsons 1993). All instruments, plastic, and glassware were periodically tested for Pb contamination by atomic absorption throughout these experiments. In addition, the accuracy of our atomic absorption technique was verified using Standard Reference Material 1486 bone meal (National Institute of Standards and Technology, Gaithersburg, MD).

### Bone Pb determination.

After 6 weeks, four animals from each of the 0, 55, 230, 2,300, and 5,800 ppm Pb groups were sacrificed. The femora and tibiae were removed, the epiphyses and soft tissues were discarded, and the bone marrow was flushed out with a 25-gauge 5/8-inch needle (Becton-Dickinson, Franklin Lakes, NJ). The remaining diaphy-seal bones were washed with phosphate-buffered saline (PBS) to remove all marrow elements and blood cells. The bones were then processed as described previously (Parsons 1993). Briefly, cortical bones were dried in a vacuum at 60°C overnight. They were then weighed dry in Teflon digestion vials (Savillex, Minnetonka, MN). Bones were wet washed with 5 mL ultrapure nitric acid 67–70% (SeaStar Chemicals, British Columbia, Canada) in the Teflon vials and taken to dryness after refluxing for 3 hr. The bone ash was then resuspended in 1 mL HNO_3_, and the total volume was brought to 10 mL with distilled water and analyzed.

### Bone marrow osteoblast differentiation.

Group B animals were sacrificed after 6 weeks of Pb exposure. The femora and unfractured tibiae were excised, and the soft tissues were removed. Primary bone marrow cells were isolated and prepared as described previously (Franzoso et al. 1998; Zhang et al. 2002). Bone marrow cells were cultured in α-minimal essential medium (α-MEM) with 10% fetal bovine serum (FBS; Hyclone Laboratories, Logan, UT) and 1% penicillin/streptomycin (Gibco) for 3 days. On the 4th day, nonadherent cells were washed off. The cells were then cultured in complete osteoblast medium of α-MEM with 10% FBS, 50 μM ascorbic acid, 1% penicillin/streptomycin, and 5 mM β-glycerophosphate. The cells were plated at 2 × 10^6^ cells per 6-cm plate for nodule formation assays, and medium was changed every 3 days. After 17 days in culture, triplicate cultures were fixed with 10% formalin, rinsed in distilled water, and stained by von Kossa’s method (Sigma Chemical Co., St. Louis, MO). Total nodular area was quantified by histomorphometry.

### Osteoclast precursor isolation.

Osteoclast progenitor cells (OCPs) were prepared from splenocytes as described previously (Schwarz et al. 1997). Animals were sacrificed after 6 weeks. Spleens were removed aseptically and placed in 10 mL Dulbecco’s Modified Eagle Medium (DMEM; Gibco). The organs were homogenized and washed through a wire mesh with 10 mL DMEM supplemented with 10% FBS and 1% penicillin/streptomycin. The cells were spun at 1,600 rpm for 5 min and then resuspended in 1 mL α-MEM, after lysing erythrocytes with cold ammonium chloride.

### Osteoclastogenesis.

Splenocytes were seeded at 1.75 × 10^5^ cells/well in a 96-well plate in α-MEM supplemented with macrophage-colony–stimulating factor (M-CSF; 30 ng/mL) and receptor activator nuclear factor κB ligand (RANKL; 100 ng/mL). Fifty percent medium was added the next day, and medium was changed every other day thereafter. Cultures were incubated at 37°C for 6–7 days and then fixed and stained for tartrate-resistant acid phosphatase (TRAP) using the leukocyte acid phosphatase kit (Sigma). The number of TRAP^+^ multinucleated cells was then counted to quantify osteoclast formation as described previously (Zhang et al. 2001).

### Flow cytometry.

Surface protein staining was performed on splenocytes. After erythrocyte lysis, a single-cell suspension was incubated in DMEM with 10% FBS. Cells were harvested in PBS containing 5 mM EDTA and stained for CD11b with biotin-labeled antibodies, as described previously (Li et al. 2004). Data were acquired using a FACScalibur cytometer and analyzed with Cell Quest software (Beckton Dickenson, Bedford, MA).

### Macrophage colony-forming assay.

The *in vitro* colony-forming assay was performed as described previously (Franzoso et al. 1997). Freshly isolated splenocytes were plated at 10^5^ cells/mL in a 35-mm dish. Splenocytes were cultured in methylcellulose-based medium (StemCell Technologies, Vancouver, British Columbia, Canada) supplemented with M-CSF (30 ng/mL) for 12 days. Individual colonies, defined as > 40 cells, were then quantified under an inverted microscope. The total number of colonies represents the original number of monocyte/macrophage and osteoclast precursors (Schwarz et al. 1997).

### Bone resorption assay.

Splenocytes were seeded at 1.75 × 10^5^ cells/well on sterile 4 mm × 4 mm bovine femoral cortical bone wafers. Cells were cultured in α-MEM with 10% FBS supplemented with M-CSF (30 ng/mL), RANKL (100 ng/mL), 1% l-glutamine, 1% penicillin/streptomycin, and 1% nonessential amino acids (Gibco). Fifty percent medium was added the next day, and medium was changed every other day thereafter. After 12 days, the wafers were scraped, dried, stained with toluidine blue, and examined under a 40× objective. Wafer images were captured, and resorption pits on the wafer surface were traced to determine the total pitted area using Osteometrics software (Osteometrics, Atlanta, GA) as described previously (Childs et al. 2001).

### Fracture.

After 6 weeks of Pb exposure, mice were anesthetized by intraperitoneal injection of 100 mg/kg ketamine HCl and 15 mg/kg xylazine. After adequate sedation, the surgical site was prepared with 70% ethanol and an incision was made about the left knee. A 25-gauge 5/8-inch needle was inserted lateral to the patellar tendon and into the tibial marrow space. This needle was then removed and a 0.25-mm diameter insect pin (Fine Science Tools, Foster City, CA) was placed into the tibia. The pin was trimmed proximally at the level of the tibial plateau. The wound was then closed with 4–0 nylon sutures. The left tibiae were placed in a modification of the guillotine three point-bending device described by Bonnarens and Einhorn (1984) and Hiltunen et al. (1993). The tibial diaphyses were fractured, without trauma to the overlying skin, using a force exerted by a 540 g weight dropped 16.5 cm. Radiographs characterized the fracture and confirmed intramedullary fixation. Radiographs were obtained using a Faxitron cabinet X-ray system (Faxitron, Wheeling, IL). The radiographic exam was repeated at 7-day intervals to follow healing and confirm maintenance of fixation.

### Histology.

Fractured tibiae were harvested at 7, 14, and 21 days. The tissues were fixed in 10% formalin for 24 hr and decalcified in 10% EDTA at pH 7.2 for 2 weeks. Samples were then processed, embedded in paraffin, and cut in 3-μm sagittal sections. Three contiguous sections (100 μm apart) for each specimen were stained with Alcian blue and counterstained with hematoxylin, orange G, and eosin (ABH/OG) as described previously (Zhang et al. 2002). Additional contiguous sections were stained for TRAP activity using naphthol AS-BI phosphate and counter-stained with hematoxylin (Sigma).

### Fracture histomorphometry.

Quantitative analysis was performed on the sagittal ABH/OG- and TRAP-stained sections by histomorphometric analysis using Osteometrics software. Bone and cartilage formation was quantified on ABH/OG-stained slides by outlining the perimeter of the fracture callus under the 2× objective. Areas of new woven bone and cartilage were then traced. This procedure was repeated until the entire fracture callus had been evaluated. The areas of woven bone, cartilage, and total fracture callus were obtained directly in square millimeters from the software. The amount of mesenchymal tissue was then calculated by subtracting bone and cartilage area from the total callus area. Bone, cartilage, and mesenchymal tissue areas each were expressed as a percentage of total fracture callus. Osteoclast quantification was performed on the TRAP-stained slides. Using the 10× objective, the perimeter of fracture callus was outlined and individual TRAP positive osteoclasts were identified and counted. The entire fracture callus was evaluated. The number of TRAP^+^ osteoclasts was then expressed per total area of fracture callus.

### In situ *hybridization.*

Plasmids corresponding to osteocalcin, collagen type II (Col II), and collagen type X (Col X) were used to synthesize ^35^S-labeled sense and anti-sense riboprobes as described previously (Ferguson et al. 1999; Zhang et al. 2002). Cut sections were incubated in hybridization buffer (50% formamide, 0.3 M NaCl, 20 mM Tris HCl, 5 mM EDTA, 10% dextran sulfate, 0.02% Ficoll, 0.02% bovine serum albumin, 0.02% polyvinyl pyrrolidone, and 0.5 mg/mL yeast RNA) containing each riboprobe at 10,000 cpm/μL, and hybridization was performed at 55°C overnight. Nonspecifically bound probe was hydrolyzed with RNase A (20 μg/μL) and washed at high stringency at 55°C with 2× salt sodium citrate/50% formamide (Ferguson et al. 1999). Emulsion-dipped slides were exposed to beta emissions for 14 days.

### Statistics.

Data are expressed as mean ± SEM. Statistical significance was determined using the Student *t*-test and analysis of variance (ANOVA) where appropriate. A value of *p* ≤0.05 was accepted as statistically significant.

## Results

### Pb exposure and whole-blood/bone Pb level determination.

**Figure 1:**
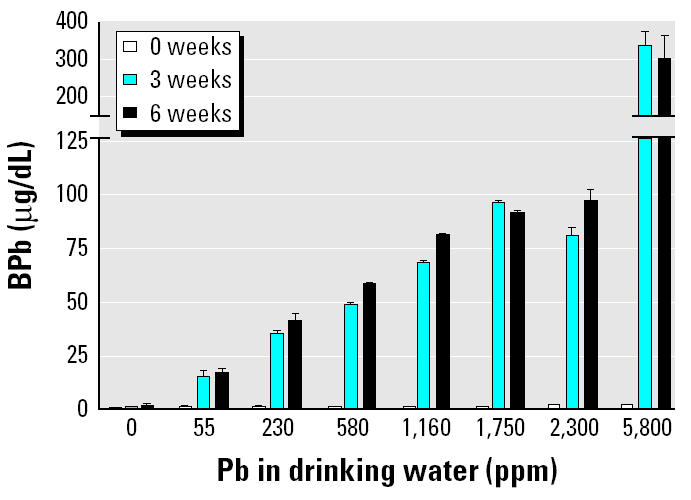
*In vivo* Pb exposures in group A mice (*n* = 4/group). Mice were exposed continuously to Pb in drinking water for the indicated time; see “Materials and Methods” for details. Error bars indicate SE.

We established a reproducible murine Pb dosing protocol by introducing various concentrations of Pb into the drinking water of group A animals and determining BPb levels, similar to our rat exposure protocol (Cory-Slechta et al. 1997). We noted that BPb levels are quite variable after short-term exposure (1–2 weeks), whereas treatments from 3 to 6 weeks resulted in stable levels (Figure 1). To assure that this treatment resulted in soft and hard tissue exposure, we determined the Pb content in blood and bone (Table 1). These data indicate that oral dosing leads to environmentally relevant exposures (10–40 μg/dL) and that BPb measurements faithfully reflect organ exposure *in vivo*. Additionally, mice appear to be very tolerant to these Pb exposures. No gross physical or behavioral changes were noted in animals with BPb levels three to four times greater than a lethal human exposure, in preliminary experiments. There was no statistically significant difference by ANOVA testing in body weight among the various dose groups in unpublished preliminary experiments (data not shown).

### Pb inhibits fracture healing.

**Figure 2:**
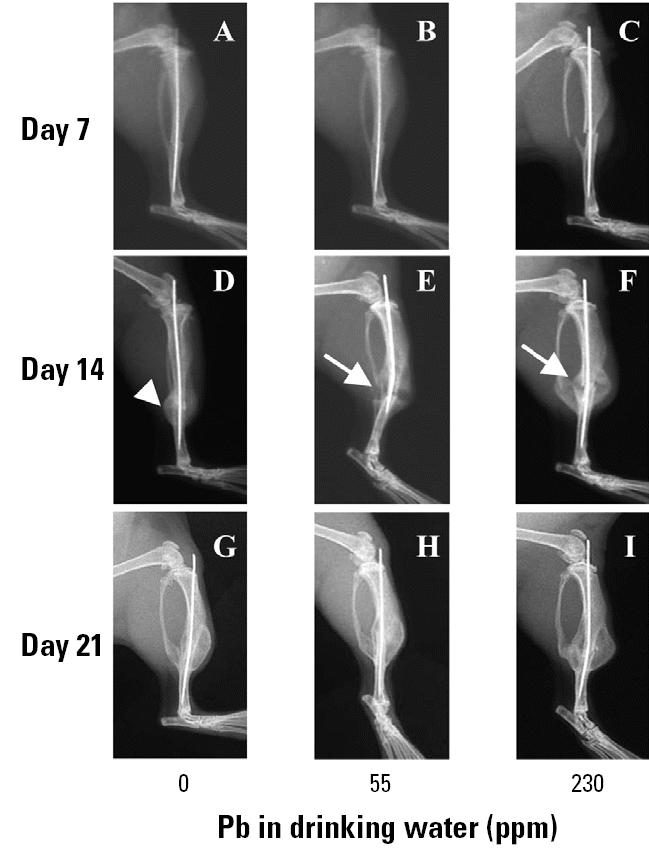
Radiographic analysis of the Pb effects on fracture healing in group B mice (*n* = 6/group). Mice were continuously exposed to Pb in drinking water for 6 weeks, with X rays from representative mice taken at the indicated time after fracture. See “Materials and Methods” for details. Arrows indicate the radiolucency in the day-14 fracture callus of Pb-treated mice (*E*, *F*), which is absent in the unexposed animals (*D*).

By 14 days after fracture, all animals showed radiographic evidence of fracture callus formation (Figure 2). However, radiographs of Pb-exposed animals demonstrated a marked increase in radiolucency within the fracture callus compared with controls (Figure 2D–F). At 21 days, all groups showed evidence of remodeling of the fracture callus with no remarkable difference in the three groups (Figure 2G–I).

**Figure 3:**
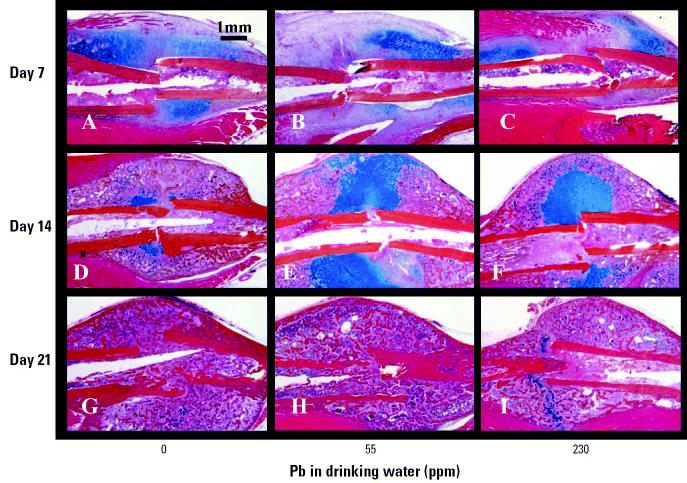
Histologic analysis of the Pb effects on fracture healing in group B mice continuously exposed to Pb in drinking water for 6 weeks (*n* = 6/group). See “Materials and Methods” for details. ABH/OG histology sections are shown at 10× magnification. Note the large amount of Alcian blue–stained cartilage in the day-14 fracture callus of Pb-treated mice (*E*, *F*). The immature fracture callus has less cartilage and exhibits a more advanced stage of remodeling in the unexposed animals (*D*).

**Figure 4:**
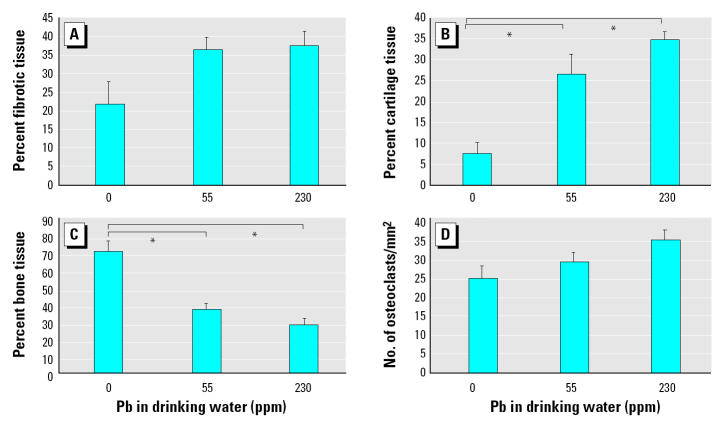
Histomorphometry of the fracture callus of group B mice continuously exposed to Pb in drinking water for 6 weeks (*n* = 6/group). See “Materials and Methods” for details. No significant differences were found between the groups at 7 and 21 days (data not shown) or between the amount of fibrotic tissue (*A*) and osteoclast numbers (*D*) between the groups. However, Pb significantly increased the amount of cartilage (*B*) and decreased the amount of bone (*C*) present in the day-14 fracture calluses of exposed mice. Error bars indicate SE.
*p < 0.05 determined using ANOVA.

Consistent with our radiographic findings, histologic analysis revealed no remarkable differences at 7 and 21 days because all fracture sites consisted of undifferentiated mesenchyme and fibrocartilage at the early time point and remodeling bone at the latter time point (Figure 3). In our day 14 untreated animals, we see the normal pattern of newly formed bone through a cartilage intermediate, as evidenced by the areas of Alcian blue–stained cartilage throughout the callus (Figure 3D). However, in the 14-day Pb-treated groups, immature cartilage accounts for the large radiolucency identified by X ray (Figure 3E–F). Histomorphometry of the day 14 fracture calluses showed a significant delay in endochondral ossification because the Pb-treated mice had a 4- to 5-fold increase in unmineralized cartilage with a commensurate decrease in bone (Figure 4). Interestingly, there was a nonlinear response to the effects of Pb on bone tissue because a similar effect was seen in both the 55 and 230 ppm treatment groups.

**Figure 5:**
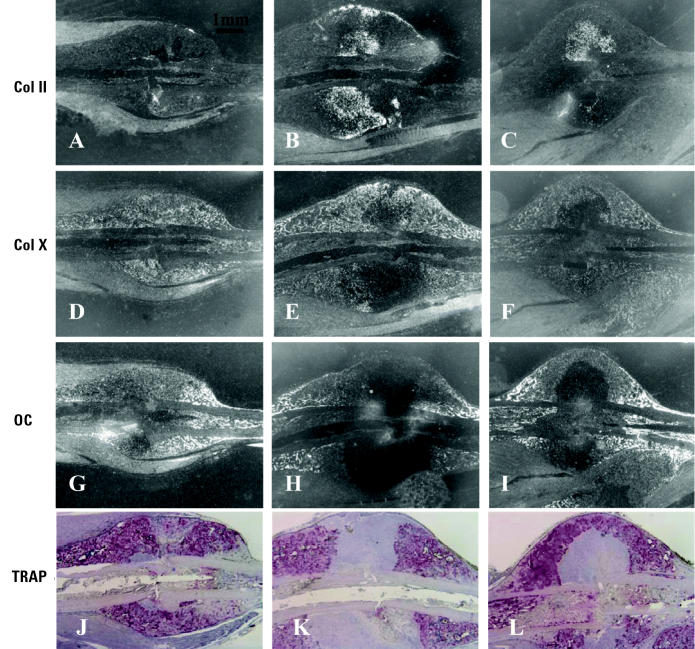
Inhibition of cartilage maturation in day-14 fracture callus of group B mice continuously exposed to Pb in drinking water for 6 weeks (*n* = 6/group). Histology sections parallel to those presented in Figure 3D–F used for *in situ* hybridization to radiolabeled antisense probes for Col II (*A–C*), Col X (*D*–*F*), or osteocalcin (OC; *G–I*) or stained for TRAP (*J–L*). There is increased Col II signal in the middle of the Pb-exposed fracture callus (*B*, *C*) compared with controls, and there is an absence of Col X (*E*, *F*), osteocalcin (*H*, *I*), and TRAP (*K*, *L*) signal in this same region.

*In situ* hybridization and TRAP staining in the 14-day fracture group helped assess phenotypic gene expression and quantify osteoclasts in the fracture callus, respectively (Figure 5). Robust expression of Col II, decreased expression of Col X, and absence of both the mature osteoblast marker osteocalcin and TRAP^+^ osteoclasts were noted in Pb-treated animals. This confirmed the prevalence of immature cartilage in the fracture callus. Importantly, gene expression outside of this immature cartilage was indistinguishable from untreated controls (Figure 5A–I), as was osteoclast number (Figures 4 and 5J–L). Thus, low Pb exposures did not completely inhibit any process of fracture healing. Rather, Pb delayed endochondral ossification.

**Figure 6:**
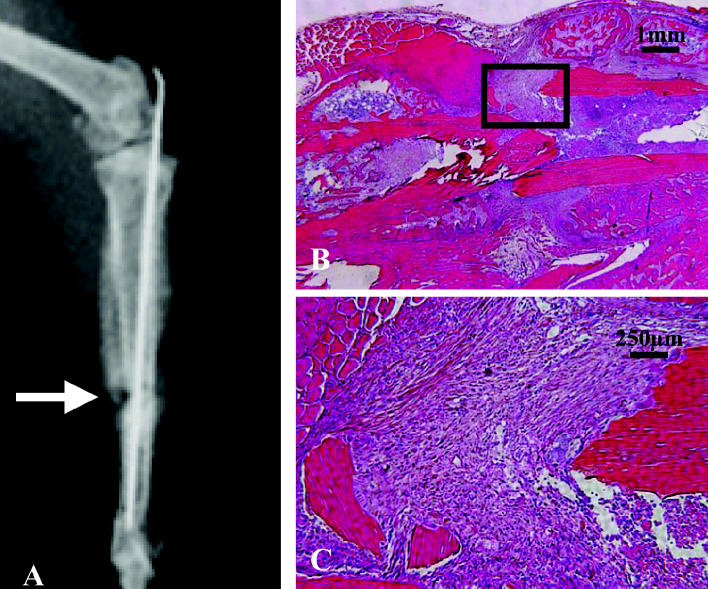
Pb exposure in group A mice (*n* = 4) exposed to 2,300 ppm Pb for 6 weeks, fractured, and assessed for skeletal repair. See “Materials and Methods” for details. A day-14 X ray from a representative mouse demonstrates the limited radiographic healing (arrow) in these mice (*A*). ABH/OG-stained histology section of day 21 fracture callus from a representative mouse at 10× (*B*) and 40× (*C*) magnification confirms the presence of fibrotic tissue between the fractured ends of the tibia and the complete absence of endochondral bone formation. These findings indicate a fibrous nonunion.

A cohort of group A mice (*n* = 4) were exposed to 2,300 ppm Pb and analyzed radiographically and histologically. In contrast to the lower-dose treatment groups (group B), the radiolucency in the day-14 X rays was not accompanied by surrounding fracture callus (Figure 6A). Furthermore, histology failed to identify evidence of endochondral ossification at the fracture site. Day 21 specimens confirmed fibrous nonunions in 75% of the group A 2,300 ppm animals (Figure 6B–C). Thus, Pb can completely inhibit fracture healing at very high doses.

**Figure 7:**
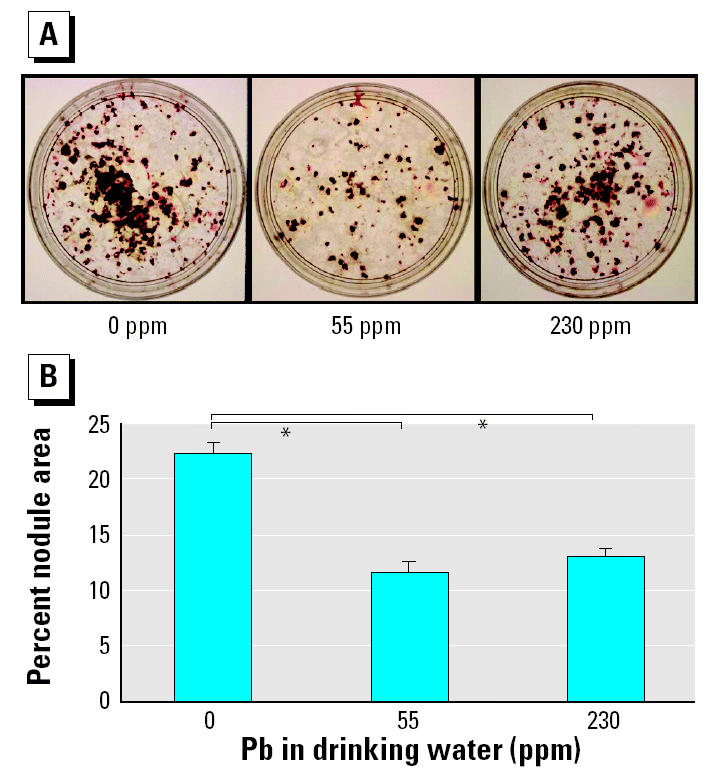
Inhibition of osteoprogenitor cells in group B mice (*n* = 6/group) continuously exposed to Pb in drinking water for 6 weeks. See “Materials and Methods” for details. (*A*) Representative photographs of the von Kossa–stained plates. (*B*) Bone nodules in these plates quantified as the percentage of nodule area as described in “Materials and Methods.”
*p < 0.05 determined using ANOVA.

The direct effects of Pb on chondrocytes (Zuscik et al. 2002), osteoclasts (Bonucci et al. 1983), and osteoblasts (Klein and Wiren 1993; Long et al. 1990; Pounds et al. 1991) have been previously documented. Our experiments on cells isolated from Pb-exposed animals focus on the mechanism by which Pb inhibits fracture healing by determining its effects on progenitor cells. When osteoprogenitor cell differentiation was examined using a bone nodule formation assay, von Kossa staining revealed that Pb-treated animals produced significantly fewer nodules at both the 55 ppm and 230 ppm dosing regimens (Figure 7). Because there was no Pb exposure in the culture medium during osteoblast differentiation, we interpret these results as a reduction in the total number of osteoprogenitor cells.

**Figure 8:**
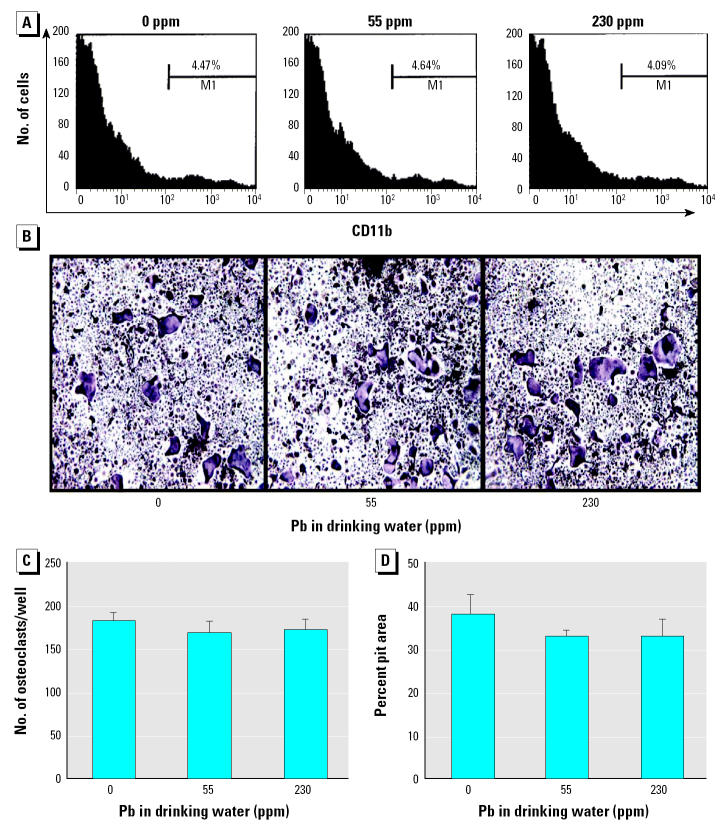
Lack of effects on osteoclast precursors after *in vivo* Pb exposure. M1, gate used to distinguish between CD11b-positive and -negative cells on the FACScalibur cytometer. Splenocytes from group B mice (*n* = 6/group) were used to determine the CD11b^+^ OCP frequency by flow cytometry analysis (*A*) or cultured in M-CSF and RANKL to form osteoclasts on tissue culture plates (*B*, *C*) or on cortical bone wafers (*D*). We observed no significant differences in OCP frequency (*A*), TRAP-stained osteoclast morphology at 10× magnification (*B*), TRAP^+^ multinucleated osteoclast formation (*C*), or bone-resorbing potential (*D*) between groups.

We found no effect on OCP number or function from *in vivo* Pb exposure. Flow cytometry analysis on splenocytes to determine the CD11b^+^ OCP frequency showed no significant Pb effects on the number of OCPs (Figure 8A). By culturing these cells with RANKL and M-CSF, we were also able to show that their potential to form osteoclasts (Figure 8B–C) and resorb bone (Figure 8D) was not affected by *in vivo* Pb exposure. These results are consistent with the fracture callus histology and indicate that *in vivo* Pb has differential effects on mesenchymal versus myeloid progenitors.

## Discussion

Toxicity due to Pb exposure remains a major public health concern and presents a broad spectrum of pathologies in children and adults. Over the last decade, our group has focused on the effects of Pb on bone and cartilage and its potential role in osteoporosis (Campbell et al. 2004; Puzas et al. 2004). Because the clinical manifestation of osteoporosis is fracture and because fracture healing is a proven model for examining cellular and molecular aspects of skeletal repair, we evaluated the effects of *in vivo* Pb exposure on callus formation, maturation, and remodeling. In addition to osteoporosis, Pb exposure is known to contribute to dental caries (Moss et al. 1999) and complications of skeletal growth in children (Kafourou et al. 1997) via poorly defined mechanisms. As such, elucidation of the Pb effects on angio-genesis, stem cell recruitment, endochondral ossification, osteoclastogenesis, and bone remodeling during fracture healing could also have implications for these other human conditions. Additionally, individuals who have high-injury-risk occupations and are concurrently exposed to Pb may also be susceptible to fracture nonunions.

Our first aim was to develop a reproducible dosing regimen that could achieve stable BPb levels over time. For this study we chose < 3, 15, and 40 μg/dL in whole blood as our targets of human background, intermediate, and high Pb exposures, respectively. We found a tight correlation between the drinking water concentration and BPb levels (Table 1) that was similar to our observations in rats (Cory-Slechta et al. 1997). We verified the incorporation of Pb into hard and soft tissues (Figure 1B), which revealed similar concentrations to those previously reported in bone (Pounds et al. 1991). Of interest, no gross toxic effects of very high BPb exposures (~ 400 μg/dL) were noted. Because this is three to four times the lethal dose in humans, future studies using murine models with human extrapolation may need to consider higher dosing.

In this study, remarkable effects of Pb on fracture healing were clearly apparent, even at the lowest dose (Figures 2–5). The phenotype can be described as an increase in chondrogenesis and delay in endochondral maturation, vascular invasion, and resorption. Although fracture healing is a highly ordered biologic process that requires precise temporal and spatial regulation, it is known that various compensatory mechanisms have evolved to rescue healing under adverse conditions. In our experience with various drug treatments and genetically deficient strains, essentially all closed stable fractures heal in mice (Flick et al. 2003; Zhang et al. 2002). Thus, the finding that the profound Pb-induced phenotype on day 14 is completely resolved by day 21 is not surprising. However, the presence of fibrous nonunions in three out of four mice with very high Pb exposure is remarkable given that all other mice studied (*n* > 100) healed. Because it is well known that mice have an extremely robust ability to heal fractures, we speculate that mice healed fractures at Pb exposures at which humans could not.

Although the precise mechanism by which Pb inhibits fracture-healing remains a topic for future investigation, the phenotype is very reproducible and is somewhat reminiscent of phenotypes described in other mouse models. The increased chondrogenesis observed is similar to that seen in the fracture callus of parathyroid hormone and prostaglandin-treated mice, indicating that Pb may be an agonist of protein kinase A signaling in chondrocytes, as predicted in our *in vitro* studies (Zuscik et al. 2002). The literature provides three potential explanations for the persistence of cartilage in the fracture callus. The first is a defect in chondrocyte apoptosis, as seen in mice with defective tumor necrosis factor receptors (Gerstenfeld et al. 2001). The second is a defect in vascular invasion of the cartilage, as seen in the matrix metalloproteinase-9 knockout mice (Colnot et al. 2003). The third is the inhibition of mesenchymal stem cell differentiation into osteoprogenitors, as seen in mice deficient in cyclooxygenase-2 (Zhang et al. 2002), which is required for callus mineralization. Evidence for this mechanism is supported by our finding that Pb-treated animals have a significant decrease in osteoprogenitor frequency (Figure 7). An additional possibility is inhibition of osteoclastic resorption and remodeling of the fracture callus, as seen in mice with defective M-CSF and RANKL signaling. However, our findings that *in situ* osteoclast numbers (Figures 4, 5) and OCP frequency (Figure 8) are unaffected by *in vivo* Pb exposure renders this possibility less likely.

The overall clinical significance of Pb inhibition of fracture healing relates to persons with osteoporosis. We have argued that because of the high environmental Pb exposures from the 1940s to 1960s, women currently going through menopause are at an additional risk of osteoporosis (Puzas et al. 2004). It is now well recognized that factors released from bone during resorption, such as TGF-β, can act on cells in the bone marrow to induce the production of osteoclastic stimulating factors or to inhibit osteoblastic new bone formation (Evans et al. 1989; Yin et al. 1999). As a consequence of the high bone turnover, which would release Pb from its inactive state in bone hydroxyapatite crystals, an additional imbalance of bone resorption over formation would occur from Pb’s preferential toxic effects on osteoblasts. Our results indicate that osteoporotic, Pb-exposed patients may sustain a fragility fracture earlier and heal their fractures at a slower rate compared with non-Pb-exposed osteoporotic individuals. Future investigations into the molecular mechanisms of Pb effects on osteoporosis and fracture healing are warranted.

## Figures and Tables

**Table 1:** *In vivo* Pb bone exposures.

Pb in drinking water (ppm)	μg Pb/g dry bone
0	0.08 ± 0.01
55	33.3 ± 2.80
230	117.3 ± 11.13
2,300	472.88 ± 61.98
5,800	682.41 ± 142.75

*n* = 4/treatment group. Data presented are mean ±SD.
